# Molecular Genetics and Pathogenesis of Ehlers–Danlos Syndrome and Related Connective Tissue Disorders

**DOI:** 10.3390/genes11050547

**Published:** 2020-05-13

**Authors:** Marco Ritelli, Marina Colombi

**Affiliations:** Division of Biology and Genetics, Department of Molecular and Translational Medicine, University of Brescia, 25123 Brescia, Italy; marco.ritelli@unibs.it

**Keywords:** Ehlers–Danlos syndrome, heritable connective tissue disorders, differential diagnosis, next generation sequencing (NGS), transcriptomics, integrated omics approaches

## Abstract

Ehlers–Danlos syndromes (EDS) are a group of heritable connective tissue disorders (HCTDs) characterized by a variable degree of skin hyperextensibility, joint hypermobility and tissue fragility. The current EDS classification distinguishes 13 subtypes and 19 different causal genes mainly involved in collagen and extracellular matrix synthesis and maintenance. EDS need to be differentiated from other HCTDs with a variable clinical overlap including Marfan syndrome and related disorders, some types of skeletal dysplasia and cutis laxa. Clinical recognition of EDS is not always straightforward and for a definite diagnosis, molecular testing can be of great assistance, especially in patients with an uncertain phenotype. Currently, the major challenging task in EDS is to unravel the molecular basis of the hypermobile EDS that is the most frequent form, and for which the diagnosis is only clinical in the absence of any definite laboratory test. This EDS subtype, as well as other EDS-reminiscent phenotypes, are currently investigated worldwide to unravel the primary genetic defect and related pathomechanisms. The research articles, case report, and reviews published in this Special Issue focus on different clinical, genetic and molecular aspects of several EDS subtypes and some related disorders, offering novel findings and future research and nosological perspectives.

Ehlers–Danlos syndromes (EDS), with an estimated prevalence of about 1/5000, belong to the large group of heritable connective tissue disorders (HCTDs) and are characterized by a variable degree of skin hyperextensibility, joint hypermobility (JHM), and tissue fragility. The clinical and genetic heterogeneity of these conditions has long been recognized, but the subjective interpretation of some semiquantitative clinical signs, such as skin hyperextensibility, skin texture, JHM, tissue fragility and bruising, led to diagnostic ambiguity and confusion regarding the type of EDS and the inclusion of similar phenotypes under the broad diagnosis of EDS. With more systematic research on clinical data and with the clarification of the molecular basis and associated pathomechanisms of several of these EDS phenotypes, different classification systems have been formulated in the past 50 years. A first classification with five main types was introduced in 1970 by Beighton [1], followed by the Berlin classification with 11 types [2], and the Villefranche nosology of 1997, in which six EDS types were included [3]. The rapid development of genetic techniques has allowed the recognition of many distinct disorders that, while dissimilar from the initially-described classic EDS types, have been given the umbrella term of EDS as an image of the presence of generalized connective tissue fragility. Hence, the last version of the EDS classification published in 2017, which recognized 13 types with 19 different causal genes mainly involved in collagen and extracellular matrix (ECM) synthesis and maintenance, has been expanded to include a wide range of clinically heterogenous disorders [4]. It should be noted that in an important percentage of EDS patients, no pathogenic variant in any of the known EDS-associated genes is identified. Therefore, it is expected that by taking advantage of next generation sequencing (NGS) technologies, further EDS types will be molecularly defined, thus demanding updating of the existing classification.

It always has been, and still is, a challenge to classify single patients in one of the existing EDS subtypes because the currently defined clinical criteria remain relatively unspecific. Often it is not possible to reach a clinical diagnosis and, therefore, the identification on molecular genetic testing of a clear pathogenic variant in a specific gene can be of great assistance, especially in patients with a clinical presentation that does not completely fit into one of the existing subtypes. The difficulty of clinical diagnosis is, among other reasons, due to the clinical overlap not only between many of the EDS subtypes but also with other HCTDs, such as Marfan, Loeys–Dietz, and arterial tortuosity syndromes, as well as some types of skeletal dysplasia and cutis laxa [4,5,6,7,8,9,10].

The major challenging task in EDS today is to unravel the molecular basis of the most frequent EDS type, namely hypermobile EDS (hEDS), the diagnosis of which remains reliant on clinical findings for the absence of any definite laboratory test. This EDS subtype as well as other EDS-reminiscent phenotypes are currently investigated worldwide to unravel the primary genetic defect and related pathomechanisms. In 2018, the groundbreaking “Hypermobile Ehlers Danlos Genetic Evaluation” (HEDGE) was launched by the International EDS Society (https://www.ehlers-danlos.com). There has never been such a worldwide collaborative effort before dedicated to discovering the underlying genetic markers for hEDS. Until the end of 2020, the HEDGE study aims to recruit, screen and undertake NGS on 1000 individuals who have been diagnosed with hEDS by the most recent clinical criteria established in 2017, which are stricter than the Villefranche criteria, in order to form homogeneous cohorts for research purposes [4]. Understanding the genetic causes of hypermobile EDS is undeniably central to the EDS community, since it will allow us to make unequivocal diagnoses for a huge number of patients. Furthermore, understanding the genetic pathways and etiopathomechanisms leading to hEDS will advise the search for possible therapeutic approaches for this disorder.

The original research articles, case report and reviews published in this Special Issue focus on different clinical, genetic, biological and molecular aspects of several EDS subtypes and some related disorders. When the first edition of McKusick’s book entitled “Heritable disorders of connective tissue” was published in 1956, less than 100 manuscripts had been dedicated to EDS; they were mainly case reports [11]. Nowadays, the search term “Ehlers–Danlos syndrome” in PubMed yields more than 4000 papers including the 14 contributions of this Special Issue, demonstrating how significant and fertile scientific research is for rare genetic diseases such as EDS and related HCTDs.

The relevance of clinical and genetic research in the continuous definition of new EDS types is exemplified in the original research article by Ritelli et al. [12], who describe a novel patient with the classical-like EDS type 2 (clEDS 2) that is caused by recessive variants in *AEBP1*, and review the clinical and molecular findings of the few patients reported to date. This rare EDS type in differential diagnosis with the more frequent classical EDS (cEDS) is not yet included in the current EDS classification, since the first description was noted in 2018 [13], but certainly it will be incorporated in the forthcoming revision of the EDS nosology. Two original research articles, respectively by Rymen et al. [14] and Micale et al. [15], further define the phenotype of the other classical-like EDS type (*TNXB* deficiency) by reporting three novel patients and performing a literature review. The authors highlight that clEDS 1 is likely underdiagnosed due to the complex structure of the *TNXB* locus which complicates diagnostic molecular testing. Rymen et al. [14] also provide an in vitro characterization of the clEDS 1 cellular phenotype, demonstrating the disorganization of the type I, III and V collagen ECMs in patient’s fibroblasts. The case report by Angwin et al. [16] underscores the importance of molecular analysis for a definite cEDS diagnosis by showing that patients with pathogenic *COL5A1* variants can have an absence of collagen flowers on skin biopsy transmission electron microscopy (TEM) analysis, which for many years has been recommended as a first line of investigation to confirm or exclude a cEDS diagnosis. The original research article of Miller et al. [17] provides novel clinical and instrumental findings on hEDS, cEDS, and vascular EDS (vEDS) by performing the assessment of pulse wave velocity measurement in a large patient cohort, which is recognized as a gold standard for determining the stiffness of arteries. The authors evidenced an increased arterial elasticity in all EDS subtypes that was associated with lower supine and seated systolic and diastolic blood pressure, thus likely contributing to the orthostatic symptoms frequently encountered in EDS, especially hEDS.

The paper by Chiarelli et al. [18] offers a wide overview on molecular mechanisms likely involved in cEDS, vEDS, and hEDS that could direct future studies to possible therapeutic strategies. The authors review their previous transcriptome and protein studies on patient dermal fibroblasts, emphasizing that these cells, despite sharing a common ECM remodeling, show differences in the underlying pathomechanisms. In cEDS and vEDS fibroblasts, key processes such as collagen biosynthesis/processing, protein folding quality control, endoplasmic reticulum (ER) homeostasis, autophagy, and wound healing emerged as perturbed. In hEDS cells, gene expression changes related to cell–matrix interactions, inflammatory/pain responses, and the acquisition of an in vitro pro-inflammatory myofibroblast-like phenotype seem to contribute to the complex pathogenesis of this molecularly unsolved EDS type. The evidence that the application of untargeted general omics approaches may serve as a valuable tool to identify novel proteins or pathways involved in the pathogenesis of the different EDS types is also documented in the original research article by Lim et al. [19] that focuses on the very rare kyphoscoliotic EDS (kEDS) type, which groups two clinically indistinguishable disorders caused by biallelic variants in either *PLOD1* or *FKBP14.* This article also proves that nowadays it is possible to perform a high-profile scientific investigation for very rare genetic disorders which have been neglected for too long. The authors performed transcriptome profiling by RNA sequencing of kEDS patient-derived skin fibroblasts that revealed the differential expression of genes encoding ECM components that are unique between *PLOD1*-kEDS and *FKBP14*-kEDS, as well as genes involved in inner ear development, vascular remodeling, ER stress and protein trafficking that were differentially expressed in patient cells compared to controls, addressing possible pharmacological targets to improve disease symptoms.

We recommend reading two papers of the Special Issue together, namely first the research article by Caraffi et al. [20] and then the paper by Ritelli et al. [21], since they present stimulating results offering nosological viewpoints concerning the so-called linkerophaties (LKs), which are caused by defects in genes involved in the glycosaminoglycan (GAG) biosynthesis. Specifically, LK genes encode for enzymes that add GAG chains onto proteoglycans via a common tetrasaccharide linker region. LKs include two different subtypes of the spondylodysplastic EDS (spEDS type 1 and 2) and further related disorders that are characterized by a variable mixed phenotype with signs of EDS and skeletal dysplasia. Of note, some of these conditions are in fact included either in the 2017 EDS classification [4] or in the 2019 nosology of skeletal dysplasia [10]. In the original research article by Caraffi et al. [20], the clinical and molecular findings of three spEDS patients are reported. Through the description of one patient with *B4GALT7*- and two patients with *B3GALT6*-spEDS and a review of previous literature reports, the authors contribute to a more accurate definition of the clinical features associated with these rare conditions. Ritelli et al. [21] report on a patient fulfilling the diagnostic criteria for spEDS according to the 2017 nosology, in whom, however, NGS identified compound heterozygosity for two pathogenic variants in *B3GAT3* that is not recognized as an EDS-causing gene. The authors review the spectrum of *B3GAT3*-related disorders and provide a comparison of all LK patients reported at the time of writing, corroborating the notion that LKs are a phenotypic continuum bridging EDS and skeletal disorders. Following these papers, we suggest reading the research article by Kumps et al. [22] that further accentuates the existing nosological confusion concerning these very rare syndromes with huge clinical overlap. Indeed, the authors describe four patients with recessive variants in *SLC39A13* that are associated with spEDS type 3, even if the encoded gene product, namely a putative zinc transporter (contrariwise to the proteins encoded by *B4GALT7* and *B3GALT6*), is not involved in GAG biosynthesis. Given that the clinical presentation of this condition in childhood consists mainly of short stature and characteristic facial features, the authors propose that the differential diagnosis is not necessarily that of a connective tissue disorder and that *SLC39A13* should be included in gene panels designed to address dysmorphism and short stature. In the outstanding review by Kosho et al. [23], the authors discuss recent advances in the pathophysiology of the rare musculocontractural EDS (mcEDS), which is caused by biallelic variants in *CHST14* and *DSE* (which are also involved in GAG synthesis). By describing novel glycobiological, pathological, and animal model-based findings, the authors highlight the critical roles of dermatan sulfate (DS) and DS-proteoglycans in the multisystem development and maintenance of connective tissues and provide fundamental evidence to support future etiology-based therapies.

Finally, three papers deal with different HCTDs in differential diagnosis with EDS. Beyens et al. [24] describe the clinical and molecular characteristics of two novel and 32 previously reported patients with occipital horn syndrome (OHS), previously known as EDS type IX or X-linked cutis laxa [2], caused by pathogenic variants in *ATP7A* (encoding a copper transporter). The main clinical features of OHS, such as cutis laxa, bony exostoses and bladder diverticula, are attributed to defective ATP7A trafficking and decreased activity of lysyl oxidase, a cupro-enzyme involved in collagen crosslinking, in line with a pathogenetic scheme shared with many EDS types. The authors explored the pathomechanisms of OHS by performing TEM analysis on skin biopsies and collagen biochemical analysis on fibroblast cultures that showed increased collagen diameter, elastic fiber abnormalities and multiple autophagolysosomes. Fusco et al. [25] report two unrelated individuals with Marfan syndrome (MFS) and Mitral valve–Aorta–Skeleton–Skin (MASS) syndrome, respectively, which were associated with different intronic variants in *FBN1*, pointing out the importance of intronic sequence analysis and the need for integrative functional studies in the diagnosis of MFS and related disorders. Camerota et al. [26] report on a cohort of 34 patients with Loeys–Dietz syndrome (LDS) with a defined molecular defect either in *TGFBR1*, *TGFBR2*, *SMAD3,* or *TGFB2*. The study broadens the clinical and molecular spectrum of LDS, corroborates and expands previously delineated genotype–phenotype correlations, and shows that a phenotypic continuum emerges as more patients are described, paving the way for a gene-based classification of the different disease subtypes.

In conclusion, this Special Issue, by offering novel findings and future research perspectives, will be of interest not only to a wide range of investigators but also to patients with EDS and related disorders, as well as to all healthcare practitioners who may encounter such syndromes during their work and we hope they will enjoy reading it.
